# Data relating to prenatal lead exposure and child IQ at 4 and 8 years old in the Avon Longitudinal Study of Parents and Children

**DOI:** 10.1016/j.neuro.2017.07.025

**Published:** 2017-09

**Authors:** Caroline M. Taylor, Katarzyna Kordas, Jean Golding, Alan M. Emond

**Affiliations:** a Centre for Child and Adolescent Health, School of Social and Community Medicine, University of Bristol, UK; b Epidemiology and Environmental Health, School of Public Health and Health Professions, University at Buffalo, Buffalo, NY, USA

**Keywords:** Child IQ, ALSPAC, Lead, Heavy metals, Pregnancy, Environmental exposure, Cognition

## Abstract

As part of the Avon Longitudinal Study of Parents and Children (ALSPAC), measures of child IQ were collected by trained psychologists. The Wechsler Pre-school and Primary Scale of Intelligence – Revised UK edition (WPPSI) was used at age 4 years in a subsample of children enrolled in ALSPAC (the Children in Focus cohort), chosen at random from the last 6 months of ALSPAC births (about 10% of the participants). At age 8 years all children enrolled in the main cohort were invited to complete a short form of the Wechsler Intelligence Scale for Children (WISC)-III ^UK^. Prenatal blood lead (B-Pb) concentrations were measured by inductively-couple plasma mass spectrometry in samples from women at a median gestation age of 11 weeks. Child blood lead was measured by atomic absorption spectrometry in samples from children attending the Children in Focus clinic at age 30 months. Maternal reports at 32 weeks’ gestation were used to generate data on a range of potential confounders. The data were used to determine the associations between prenatal exposure to lead and child IQ at 4 and 8 years. The effect of child B-Pb at 3 years as a moderator of these associations was tested. (For results, please see doi:10.1016/j.neuro.2017.07.003 Taylor et al., (2017)).

**Specifications table**

**Table:** 

Subject area	*Human Biology*
More specific subject area	*Child development*
Type of data	*Table*
How data was acquired	*Longitudinal cohort study questionnaire data, biological assessment*
Data format	*Edited and analysed*
Experimental factors	*Maternal self-completion questionnaires; maternal and child blood assays for lead; clinic assessments of child IQ*
Experimental features	*Mean IQ scores at 4 and 8 years compared with maternal prenatal lead levels and child lead levels at 3 years old*
Data source location	*Former Avon area, centred around Bristol, UK*
Data accessibility	*Data are within this article*

**Value of the data**

• The ALSPAC dataset contains information on a large number of children in a geographically defined population whose development was monitored to age 24–25 years old at present (2017)).
• The data provide a basis for early identification of adverse effects of environmental exposures (metals and other toxicants).
• The data allow detailed analyses of family and social circumstances and their associations with child development.

## 1 Data

In this paper, we describe data on child IQ at 4 and 8 years, prenatal B-Pb concentrations and child B-Pb concentrations at age 2.5 years (see Tables).

The ALSPAC study website contains details of all the data that are available through a fully searchable data dictionary:http://www.bris.ac.uk/alspac/researchers/data-access/data-dictionary/. Data can be obtained by bona fide researchers after application to the ALSPAC Executive Committee (http://www.bristol.ac.uk/alspac/researchers/access/).

## 2 Experimental design, materials and methods

### 2.1 Blood lead measurements

#### 2.1.1 Prenatal samples

Whole blood samples were collected in acid-washed heparin vacutainers (Becton and Dickinson) by midwives as early as possible in pregnancy. Midwives’ participation in collecting the bloods was voluntary, dependent on time available and consequently was only obtained in two of the three Health Authority areas of the recruitment region. Altogether 4484 samples were collected at a median gestational age of 11 weeks (range 1–42 weeks, mode 10 weeks, interquartile range 9–13 weeks). The social background of the women who gave the samples did not differ from the rest of the ALSPAC population apart from being slightly older and more educated (Taylor et al., 2013). Samples were stored at 4 °C at the collection site and then sent to the central Bristol laboratory within 0–4 days. These samples were kept at room temperature for up to 3 h during transfer, and were stored at 4 °C as whole blood in the original tubes for 18–19 years before being sent for analysis.

The method of assay of lead has been described in detail elsewhere (Taylor et al., 2013). In brief, the laboratory of Robert Jones at the Centers for Disease Control and Prevention (CDC) developed methods to prepare the samples for analysis of whole blood lead (CDC method 3009.1). Clotted whole blood was digested to remove all clots before being analysed using inductively coupled plasma dynamic reaction cell mass spectrometry (ICP-DRC-MS). Two levels of bench quality control (QC) materials, as well as a blind QC material, were used for daily quality control.

There were 4484 samples available for lead assays of which 4285 were successfully analysed (tube/vial broken n = 7, suspect sample n = 3, quantity not sufficient for repeat testing n = 67, lab error n = 122). One of the samples had a lead concentration below the limit of detection of the assay (0.24 μg/dL). For this sample, in consideration of the distribution of the lead concentrations, a value of 0.7 times the limit of detection value (limit of detection/√2) was considered to be a better estimate of the value than taking a mid-point (Hornung and Reed, 1990, Centers for Disease Control and Prevention, 2005). The mean level was 3.67 ± 1.47 (range 0.29–19.14, median 3.41) μg/dL.

#### 2.1.2 Child samples

A randomly selected sample of parents whose babies were born within the last 6 months of enrollment into ALSPAC were invited to bring their children to a research clinic (Children in Focus, CiF) at age 30 months. Parental consent for a venous blood sample was obtained from 81% of the 1135 children in the CiF group. A venous blood sample was collected in lead-free tubes from 71% (n = 653) of clinic attenders; 69 samples had insufficient volume for analysis, leaving 582 samples for analysis.

The blood lead concentration was measured at Southampton General Hospital, UK, by atomic absorption spectrometry using micro-sampling flame atomisation. Details of the quality control procedures have been published (Chandramouli et al., 2009). The mean level was 4.22 ± 3.12 (range 0.83–27.56, median 3.31) μg/dl.

### 2.2 Child IQ measurements

#### 2.2.1 IQ at age 4 years

Mental development at age 4 years was measured using the Wechsler Pre-school and Primary Scale of Intelligence – Revised UK edition (WPPSI) (Wechsler, 1990) at a research clinic for children in the CiF subsample. All cores scales were administered. The children were also given a digit span test of short term memory, devised and standardised by Professor Susan Gathercole (research psychologist).

Inter-rater reliability was ensured as follows. The testers were overseen by Steve Gibbs, a tester with long experience of psychometric testing with ALSPAC. He observed each tester, met with the group regularly to discuss the precise administration of each test, and supervised and checked their scoring. Each tester scored four videos of tests and interindividual scores were compared.

The WPPSI comprises ten subtests: five verbal and five performance. The verbal subtest scores were combined to make up the *verbal IQ*, and the performance scores combined to make up the *performance IQ*. The ten subtest scores were combined to produce a full-scale IQ score. Following each child’s session, which usually lasted 50–60 min, the parent or carer was given a short questionnaire asking whether the child’s behaviour and performances was typical, and if not, how and why.

If a child completed fewer than four subtests on the performance scale then the final performance IQ score was not calculated (and therefore not the full-scale score either). If, however, the child completed four out of the five subtests, the mean of the four subtests was calculated and imputed for the subtest not completed, so that a performance score could be computed. This *prorating* is standard WPPSI practice. Identical rules applied to the verbal score. Thus, some children, although not completing a subtest, had a score for that subtest.

#### 2.2.2 IQ at 8 years

Mental development at age 8 years was measured by the Wechsler Intelligence Scale for Children WISC-III ^UK^ (Wechsler et al., 1992) at a research clinic for all children enrolled in the ALSPAC cohort. A short form of the measure was employed, where alternate items were used for all subtests, with the exception of the coding subtest which was administered in full. Hence the length of the sessions was reduced and the children were less likely to become tired. The WISC comprises five verbal subtests (Information, Similarities, Arithmetic, Vocabulary, Comprehension) and five performance subtests (picture completion, coding, picture arrangement, block design, object assembly). The children were also given the forwards and backwards digit span task (a measure of short-term memory). The verbal subtest scores were combined to make up the verbal IQ and the performance scores were combined to make the performance IQ. The ten subtest scores were combined to produce a full-scale (total) IQ score.

Inter-rater reliability was ensured as follows: the testers were trained psychologists, who were overseen by Dr Claire Bell, a senior psychologist with long experience of psychometric testing within the study. She observed each tester, and met with the group regularly to discuss the precise administration of each subtest and checked their scoring.

The task was made as reassuring and unstressful for the child as possible, with the tester explaining that the child would be playing lots of games: looking at pictures, doing puzzles, making patterns and answering some questions. It was explained that some of the things might get quite difficult but not to worry as they were the same things we would ask older children to play. All children were encouraged to have a go at things, even if they thought they were just guessing.

Raw scores were calculated according to the items used in the alternate item form of the WISC. This was achieved by summing the individual items within each subtest and multiplying by 2 for picture completion, information, arithmetic, vocabulary, comprehension and picture arrangement; multiplying by 5/3 for similarities, multiplying by 3/2 for object assembly and block design, thus, making the raw scores comparable to those that would have been obtained had the full test been administered (the raw score for the coding subtest was calculated in the standard way as the full subtest was administered). It is because of this multiplication that some of the scores do not follow a smooth distribution.

For a small number of cases, scores could be imputed where a tester or computer error had been made and such a score would otherwise have been missing. Dr Bell made such decisions on a case by case basis.

### 2.3 Questionnaire assessments

The ALSPAC study included the distribution of questionnaire by mail to the pregnant woman for self-completion and return in a pre-paid envelope at 32 weeks’ gestation.

### 2.4 Publications

Publications on associations of prenatal exposures with child IQ in the ALSPAC cohort are shown in Table 1. Publications on associations of prenatal lead with measures of child development are shown in Table 2. Other publications using the prenatal and child lead measures are shown in Table 3.

**Table 1:** Publications using prenatal exposures and child IQ in the ALSPAC cohort.

Authors	Exposure	Outcome	Results
Alati et al. (2008)	Prenatal smoking and alcohol	IQ at 8 years	Associations of maternal smoking and alcohol consumption were similar to those for paternal smoking and alcohol consumption, suggesting effect not explained by intrauterine exposure
Bath et al. (2013)	Maternal iodine status	IQ at 8 years	Maternal mild-to-moderate iodine deficiency associated with likelihood of child being in the lowest quartile of verbal IQ
Bonilla et al. (2012a)	Prenatal plasma vitamin B_12_	IQ at 8 years	No association
Bonilla et al. (2012b)	Fasting glucose and type 2 diabetes associated genetic variants in pregnancy	IQ at 8 years	No association of maternal fasting glucose genetic risk score Positive association of maternal type 2 diabetes genetic risk score
Freitas-Vilela et al. (2017)	Dietary patterns in pregnancy	IQ at 8 years	Positive association of being in the ‘fruit and vegetable’ cluster on child IQ compared with the ‘meat and potato’ cluster and the ‘white bread and coffee’ cluster
Golding et al. (2017)	Prenatal blood mercury	IQ at 8 years	No association providing the mother consumes fish
Hibbeln et al. (2007)	Prenatal fish consumption	IQ at 8 years	Positive association at maternal fish intakes of >340 g/week
Zuccolo et al. (2013)	Prenatal alcohol	IQ at 8 years	Positive association of moderate drinking compared with light drinking on child IQ, possibly reflecting residual confounding

**Table 2:** Publications on lead exposure and measures of child development in the ALSPAC cohort.

Authors	Exposure	Outcome	Results
Chandramouli et al. (2009)	Child blood lead	Academic performance and behaviour	Negative associations with school performance tests and antisocial behaviour
Maisonet et al. (2014)	Prenatal blood lead	Puberty timing in girls	No associations with age at menarche or rate of attainment of pubertal markers
Taylor et al. (2014a)	Prenatal blood lead	Secondary sex ratio	No association with secondary sex ratio
Taylor et al. (2015a)	Prenatal blood lead	Birth outcomes	Negative associations with anthropometry at birth, mean birthweight and preterm delivery, but not low birthweight
Taylor et al. (2015b)	Prenatal and child blood lead	Balance ability at 7 and 10 years	No associations with balance ability
Taylor et al. (2016)	Prenatal blood lead	Birth outcomes	No evidence for supralinear dose–response relationship or lower limit of level of concern
Taylor et al. (2017)	Prenatal blood lead	Child IQ at 4 and 8 years	No associations with child IQ

**Table 3:** Other publications on prenatal and child lead measures in ALSPAC.

Authors	Exposure	Results
Golding et al. (1998)	Child blood lead	Documentation of child blood lead level
Taylor et al. (2013)	Prenatal blood lead	Environmental factors predicting prenatal blood lead level
Taylor et al. (2014b)	Prenatal blood lead	Documentation and review of prenatal blood levels and international levels of concern
Warrington et al. (2015)	Maternal blood lead	Association with polymorphism in *ALAD*

### 2.5 Associations with prenatal lead

In our parallel paper (Taylor et al., 2017) we show that prenatal lead exposure was not associated with adverse effects on child IQ at age 4 or 8 years in ALSPAC. There was, however, some evidence to suggest that boys are more susceptible than girls to prenatal exposure to lead. Here we show:

(i) Characteristics of ALSPAC participants included and excluded in the study (complete cases) (Table 4)

**Table 4:** Characteristics of ALSPAC participants included and excluded in the study (complete cases).

	Age 4 years			Age 8 years		
	Included	Excluded	P value	Included	Excluded	P value
Mother						
Education						
None/CSE	47 (13.5%)	2464 (20.4%)	0.004	223 (12.2%)	2288 (21.5%)	<0.001
Vocational	28 (8.0%)	1198 (9.9%)		141 (7.7%)	1085 (10.2%)	
O level	123 (35.3%)	4194 (34.6%)		600 (32.9%)	3717 (35.0%)	
A level	95 (27.3%)	2703 (22.3%)		499 (27.4%)	2299 (21.6%)	
Degree	55 (15.8%)	1549 (12.8%)		360 (19.7%)	1244 (11.7%)	
Whole life in Avon						
Yes	174 (50.0%)	6848 (53.6%)	0.192	877 (48.1%)	6145 (54.3%)	<0.001
	174 (50.0%)	5939 (46.4%)		946 (51.9%)	5167 (45.7%)	
Parity						
0	158 (45.4%)	5604 (46.4%)	0.870	839 (46.0%)	4923 (44.8%)	0.331
≥1	190 (54.6%)	6860 (53.6%)		984 (54.0%)	6066 (55.2%)	
Smoking						
No	301 (86.5%)	9432 (79.0%)	0.001	1567 (86.0%)	8166 (78.1%)	<0.001
Yes	47 (13.5%)	2501 (21.0%)		256 (14.0%)	2292 (21.9%)	
Age						
< 25	48 (13.8%)	3307 (24.2%)	<0.001	227 (12.5%)	3128 (25.6%)	<0.001
≥25–29	130 (37.4%)	5302 (38.7%)		697 (38.2%)	4735 (38.7%)	
≥30–34	125 (35.9%)	3744 (27.3%)		659 (36.1%)	3210 (26.3%)	
≥35	45 (12.9%)	1347 (9.8%)		240 (13.2%)	1152 (9.4%)	
Housing						
Mortgaged/owned	283 (81.3%)	9318 (73.1%)	0.001	1538 (84.4%)	8063 (71.3%)	<0.001
Rented/other	65 (18.7%)	3425 (26.9%)		285 (15.6%)	3205 (28.4%)	

Child						
Gestation (weeks)	39.6 ± 1.7	39.3 ± 2.1	0.009	39.5 ± 1.8	39.3 ± 2.1	0.003
Birthweight (g)	3495 ± 535	3382 ± 574	<0.001	3432 ± 568	3378 ± 573	<0.001
Sex						
Female	151 (43.4%)	9057 (50.8%)	0.064	899 (49.3%)	8309 (48.2%)	0.364
Male	197 (56.6%)	9658 (47.6%)		924 (50.7%)	8931 (51.8%)
(ii) Maternal characteristics by B-Pb ≤5 or >5 μg/dl (n (%)) (complete cases at age 8 years) (Table 5)

**Table 5:** Maternal characteristics by ≤5 or >5 μg/dl (n (%)) (complete cases at age 8 years).

	Maternal B-Pb (μg/dl)		P value
	≤5	>5	
Mother			
Education			
None/CSE	191 (12.3%)	32 (11.9%)	<0.001
Vocational	127 (8.2%)	14 (5.2%)	
O level	518 (33.3%)	82 (30.5%)	
A level	439 (28.2%)	60 (22.3%)	
Degree	279 (18.0%)	81 (30.1%)	
Whole life in Avon			
No	793 (51.0%)	153 (56.9%)	0.076
Yes	761 (49.0%)	116 (43.1%)	
Parity			
0	705 (45.4%)	134 (49.8%)	0.177
≥1	849 (54.6%)	135 (50.2%)	
Smoking			
Yes	191 (12.3%)	65 (24.2%)	<0.001
No	1363 (87.7%)	204 (75.8%)	
Age			
<25	206 (13.2%)	21 (7.7%)	0.008
≥25–29	597 (38.4%)	100 (37.2%)	
≥30–34	562 (36.2%)	97 (36.1%)	
≥35	189 (12.2%)	51 (19.0%)	
Alcohol			
Yes	407 (32.8%)	110 (55.8%)	0.002
No	832 (67.2%)	87 (44.2%)	
Housing			
Mortgaged/owned	1299 (83.6%)	239 (88.8%)	0.028
Rented/other	255 (16.4%)	30 (11.2%)	
Family adversity index			
0–5	1532 (98.6%)	260 (96.7%)	0.024
6–11	22 (1.4%)	8 (3.3%)	
Crowding index			
≤0.5	738 (47.5%)	157 (58.4%)	0.011
>0.5–0.75	507 (32.6%)	70 (26.0%)	
>0.75–1	255 (16.4%)	36 (13.4%)	
>1	54 (3.5%)	6 (2.2%)	

Child			
Gestation (weeks)	39.5 ± 1.75	39.2 ± 2.1	0.028
Birthweight (g)	3442 ± 559	3373 ± 613	0.068
Sex			
Female	756 (48.6%)	143 (53.2%)	0.172
Male	798 (51.4%)	126 (46.8%)	

Chi-square/ANOVA.
(iii) Effect sizes of selected variables in model 3 in Table 2 of the parallel paper (Taylor et al., 2017) (R^2^) (complete cases) (Table 6)

**Table 6:** Effect sizes of selected variables in model 3 in Table 2 in Taylor et al., 2017 (R^2^) (complete cases).

Variable	R^2^ (4 years)			ΔR^2^ (8 years)		
	Verbal IQ	Performance IQ	Total IQ	Verbal IQ	Performance IQ	Total IQ
Maternal Pb	0.001	0.001	0.001	0.001	0.000	0.001
Sex	**0.011**	**0.009**	**0.013**	0.001	0.004	0.000
Age at testing	0.008	0.000	0.003	0.008	**0.010**	0.009
Maternal education	**0.018**	**0.022**	**0.026**	**0.036**	**0.035**	**0.047**
Smoking	0.003	0.002	0.003	0.000	0.000	0.000
Alcohol	0.003	0.005	0.005	0.000	0.000	0.000
Age	**0.020**	0.002	**0.012**	**0.011**	0.000	0.003
Parity	**0.028**	0.003	**0.016**	0.006	0.000	0.000
Time in Avon	0.002	0.002	0.003	0.009	0.001	0.006
Housing tenure	0.003	**0.010**	0.007	0.003	0.002	0.003
Household crowding	0.001	0.006	0.003	0.001	0.000	0.001
Family adversity index	0.000	0.006	0.003	0.002	0.003	0.004
Weighted life events scores	0.001	0.006	0.003	0.000	0.000	0.000

R^2^ > 0.010 shown in bold.
(iv) Association of prenatal B-Pb >5 μg/dl with child IQ at age 4 and 8 years (logistic regression) in ALSPAC: multiple imputation (Table 7)

**Table 7:** Association of prenatal B-Pb >5 μg/dl with child IQ at age 4 and 8 years (logistic regression) in ALSPAC: multiple imputation.

Age (years)	IQ test		n	Regression analyses: Model 3	
				OR (95% CI)	P
Multiple imputation					
4	WPPSI	Verbal IQ	404	1.43 (0.63, 3.23)	0.397
		Performance IQ	404	0.99 (0.42, 2.33)	0.981
		Total IQ	404	0.91 (0.37, 2.24)	0.838

8	WISC	Verbal IQ	2217	0.72 (0.52, 1.00)	0.053
		Performance IQ	2217	1.00 (0.74, 1.35)	0.995
		Total IQ	2217	0.74 (0.53. 1.02)	0.065

Reference: highest three quartiles of IQ score elided (vs lowest IQ quartile)

See Methods for details of variables.

Model 3: adjusted for sex, actual age at testing, maternal education, smoking in pregnancy, alcohol in pregnancy, maternal age, parity, time resident in Avon, housing tenure, household crowding, family adversity index, weighted life events score.
(v) Association of prenatal B-Pb on child IQ at age 8 years by sex in ALSPAC: multiple imputation (Table 8)

**Table 8:** Association of prenatal B-Pb on child IQ at age 8 years by sex in ALSPAC: multiple imputation.

Age (years)	IQ test		IQ scores			Regression analyses: Model 3^a^					
			Boys	Girls	p	Boys			Girls		
						R^2^	Unstandardised B coefficient (95% CI)	p	R^2^	Unstandardised B coefficient (95% CI)	p
Multiple imputation											
4	WPPSI	n	230	174							
		Verbal IQ	99.2± 13.4	102.2 ± 13.2	0.022	0.192^b^	−0.01 (−1.26, 1.24)	0.989	0.205	−0.65 (−1.95, 0.65)	0.326
		Performance IQ	106.8 ± 15.2	110.7 ± 13.3	0.007	0.165^b^	0.24 (−0.20, 1.77)	0.744	0.157	−0.12 (−1.47, 1.23)	0.860
		Total IQ	103.1 ± 14.3	107.1 ± 13.5	0.005	0.220^b^	0.20 (−1.12, 1.51)	0.767	0.212	−0.48 (−1.80, 0.84)	0.479

8	WISC	n	1113	1104							
		Verbal IQ	108.1 ± 17.6	107.3 ± 16.0	0.284	0.181^b^	−0.01 (−0.68, 0.67)	0.985	0.233^b^	0.75 (0.18, 1.31)	0.009
		Performance IQ	98.7 ± 17.4	100.6 ± 16.4	0.006	0.089^b^	−0.23 (−0.93, 0.47)	0.527	0.103^b^	0.56 (−0.06, 1.19)	0.076
		Total IQ	104.2 ± 17.0	104.8 ± 15.7	0.412	0.175 ^b^	−0.12 (−0.77, 0.54)	0.727	0.221^b^	0.74 (0.19, 1.30)	0.009

See Methods for details of variables.

^a^Model 3: adjusted for sex, actual age at testing, maternal education, smoking in pregnancy, alcohol in pregnancy, maternal age, parity, time resident in Avon, housing tenure, household crowding, family adversity index, weighted life events score.

^b^R^2^ for 20th imputation.
(vi) Association of prenatal B-Pb on child IQ at age 4 years and 8 years (linear regression) in ALSPAC: multiple imputation (Table 9)

**Table 9:** Association of prenatal B-Pb on child IQ at age 4 years and 8 years (linear regression) in ALSPAC: multiple imputation.

Age (years)	IQ test			R^2^	Unstandardised B coefficient (95% CI)	P values	
						B coefficient	Sex × prenatal B-Pb interaction
Multiple imputation							
4	WPPSI (n = 404)	Verbal IQ	Model 3	0.182^b^	−0.17 (−1.06, 0.72)	0.707	0.775
		Performance IQ	Model 3	0.154^b^	0.22 (−0.76, 1.20)	0.656	0.985
		Total IQ	Model 3	0.204^b^	0.02 (−0.90, 0.94)	0.960	0.831

8	WISC (n = 2217)	Verbal IQ	Model 3	0.201^b^	0.39 (−0.05, 0.82)	0.082	0.079
		Performance IQ	Model 3	0.095^b^	0.19 (−0.28, 0.65)	0.438	0.112
		Total IQ^a^	Model 3	0.192^b^	0.33 (−0.10, 0.76)	0.127	0.061

See Methods for details of variables.

Model 3: adjusted for sex, actual age at testing, maternal education, smoking in pregnancy, alcohol in pregnancy, maternal age, parity, time resident in Avon, housing tenure, household crowding, family adversity index, weighted life events score.

a n = 1823.

b R^2^ for 20th imputation.
(vii) Effect of maternal haemoglobin in the association of prenatal B-Pb and child IQ (complete cases) (Table 10)

**Table 10:** Effect of maternal Hb in association of prenatal Pb and child IQ (complete cases).

Age (years)	IQ test			R^2^	Unstandardised B coefficient (95% CI)^a^	P value
4	WPPSI (n = 246)	Verbal IQ	Model 3	0.216	−0.65 (−1.77, 0.47)	0.254
			Model 3 plus maternal Hb	0.216	−0.63 (−1.76, 0.49)	0.269
		Performance IQ	Model 3	0.168	−0.61 (−1.94, 0.73)	0.372
			Model 3 plus maternal Hb	0.168	−0.62 (−0.96, 0.73)	0.367
		Total IQ	Model 3	0.226	−0.69 (−0.19, 0.48)	0.247
			Model 3 plus maternal Hb	0.226	−0.69 (−1.88, 0.50)	0.252

8	WISC (n = 1328)	Verbal IQ	Model 3	0.189	0.23 (−0.35, 0.80)	0.440
			Model 3 plus maternal Hb	0.189	0.25 (−0.33, 0.83)	0.394
		Performance IQ	Model 3	0.112	0.08 (−0.53, 0.68)	0.804
			Model 3 plus maternal Hb	0.112	0.10 (−0.51, 0.71)	0.743
		Total IQ^b^	Model 3	0.195	0.20 (−0.36, 0.76)	0.480
			Model 3 plus maternal Hb	0.195	0.23 (−0.33, 0.79)	0.421

**Fig. 1:**
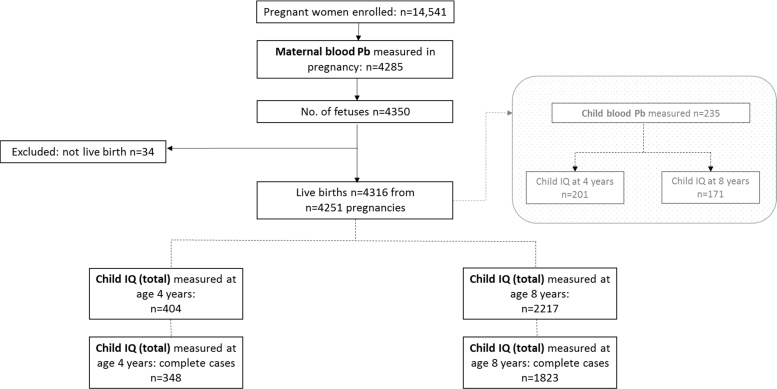
Study flow chart.

Model 3: adjusted for sex, actual age at testing, maternal education, smoking in pregnancy, alcohol in pregnancy, maternal age, parity, time resident in Avon, housing tenure, household crowding, family adversity index, weighted life events score.

a n=1823.^b^R2 for 20th imputation.
(viii) Study flow chart (Fig. 1)

## Funders

The UK Medical Research Council and the Wellcome Trust, United Kingdom (Grant ref: 102215/2/13/2) and the University of Bristol, United Kingdom currently provide core support for ALSPAC. The assays of the maternal blood samples were carried out at the Centers for Disease Control and Prevention with funding from NOAA. CMT was supported by a Wellcome Trust Career Re-Entry Fellowship (Grant ref: 104077/Z/14/Z). The funders had no involvement in the study design nor in the collection, analysis and interpretation of the data.

## Completing financial interests

The authors have no competing interests.

## Ethics approval

Ethics approval for the study was obtained from the ALSPAC Ethics and Law Committee and Local Research Ethics Committees.
